# A proteomic approach identified growth hormone-dependent nutrition markers in children with idiopathic short stature

**DOI:** 10.1186/1477-5956-6-35

**Published:** 2008-12-11

**Authors:** Gunnel Hellgren, Björn Andersson, Andreas FM Nierop, Jovanna Dahlgren, Ze'ev Hochberg, Kerstin Albertsson-Wikland

**Affiliations:** 1 Göteborg Pediatric Growth Research Center, Department of Pediatrics, Institute of Clinical Sciences, Sahlgrenska Academy, University of Gothenburg, The Queen Silvia Children's Hospital, SE-416 85 Göteborg, Sweden; 2 Muvara bv, Leiderdorp, The Netherlands; 3 Meyer Children's Hospital, Rambam Medical Center, and Technion – Israel Institute of Technology, Haifa, Israel

## Abstract

**Background:**

The broad range in growth observed in short prepubertal children receiving the same growth hormone (GH) dose is due to individual variation in GH responsiveness. This study used a pharmaco-proteomic approach in order to identify novel biomarkers that discriminate between short non-GH-deficient (GHD) children who show a good or poor growth response to GH treatment.

A group of 32 prepubertal children with idiopathic short stature (ISS) were included in the study. Children were classified on the basis of their first year growth velocity as either good (high responders, n = 13; range, 0.9–1.3 standard deviation score (SDS) or poor (low responders, n = 19; range, 0.3–0.5 SDS) responders to GH treatment (33 μg/kg daily).

Serum protein expression profiles before, and after 1 year of GH treatment, were analyzed on a weak cationic exchange array (CM10) using surface-enhanced laser desorption/ionisation time-of-flight mass spectrometry (SELDI-TOF-MS).

**Results:**

Changes in the intensity of two protein peaks (13.788 kDa and 17.139 kD) during the study period allowed the correct classification of 82% of children as high and low responders, respectively. The 13.788 kD peak, transthyretin, decreased in the high-responder group and increased in the low-responder group during 1 year of GH treatment, whereas the 17.139 kDa peak, apolipoprotein A-II (Apo A-II) decreased in the high-responder group and remained unchanged in the low-responder group. These peaks were identified by the consistency of peak pattern in the spectra, serum depletion experiments using specific antibodies and mass spectrometry.

**Conclusion:**

Our results suggest that transthyretin and apolipoprotein A-II may have a role in GH sensitivity and could be used as markers to predict which short prepubertal children with ISS will show a good or poor response to GH treatment.

## Background

Short stature is believed to be the result of many factors. These include genetics, environment and many different hormones. The growth hormone (GH)-dependent component of growth during childhood is believed to result from the integrated balance between GH secretion and GH responsiveness in each child. Short stature in GH-deficient (GHD) children arises as a result of poor GH secretion, whereas short stature in children with idiopathic short stature (ISS) may be due to differences in the degree of GH responsiveness.

As we know that GH and insulin-like growth factor I (IGF-I) play a major role in the regulation of postnatal growth, most studies investigating GH responsiveness in patients have involved assessments of mutations in single genes involved in the GH/IGF-I axis [1,2]. However, very few growth-related mutations have been identified by this approach and it is clear that other, as yet unknown, factors must be involved in responsiveness to GH treatment.

Prediction models for growth response to GH treatment, a measure of GH responsiveness, suggest a role for auxological data, such as growth during infancy and childhood [3], familial differences in height, height standard deviation score (SDS) at treatment start and differences in mid-parental height, as well as the responsiveness of the GH/IGF-I axis [4-7]. Variables used in these prediction models, however, explain only around 50–70 % of the growth response to GH.

This study explored the utility of a pharmaco-proteomic approach for the identification of novel biomarkers that allow the prediction of GH treatment response. For this purpose we monitored changes in protein profiles using surface-enhanced laser desorption/ionization time-of-flight mass spectrometry (SELDI-TOF-MS). We analyzed the expression of serum proteins before the start of GH treatment and after 1 year of treatment in prepubertal children with ISS and assessed differences in protein expression between patients with good and poor growth responses to GH treatment. By using this approach two nutrition proteins were identified that could be used to discriminate between good and poor GH treatment responders with respect to treatment response after 1 year of treatment, and to a lower degree before treatment.

## Subjects and methods

### Study population

**Figure 1:**
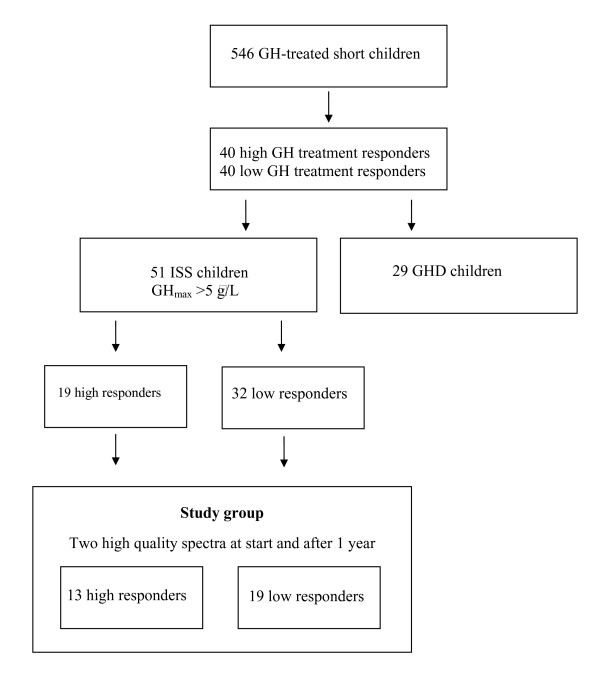
The procedure for study group selection.

The procedure for study selection is shown in Figure 1. From a group of 546 short (height below -2 SD [8]) prepubertal children receiving GH treatment, the 40 children with highest and the 40 children with lowest response on the basis of their first year growth were selected. From this group of 80 children, 51 children with ISS and a maximum peak of GH secretion (GH_max_) on the arginine-insulin tolerance test (AITT) > 5 μg/L were identified. Of this subgroup, 19 were classified as high treatment responders (first year height increase of 0.9–1.3 SDS), and 32 as low treatment responders (0.1–0.5 SDS). Only patients for whom two high quality mass spectra measurements were available at each time point were included in further statistical analysis. The final study population included 37 children (14 high responders; 23 low responders) for whom protein expression data were available before the start of GH treatment and 32 children (13 high responders; 19 low responders) for whom protein expression data were available after 1 year of GH treatment.

Auxological data for children for whom two high quality spectra were available both before treatment and after 1 year are presented in Table 1.

**Table 1:** Auxological data for the children included in the analysis, from whom high-quality spectra were available both before and after 1 year of GH treatment.

	**High-responder group**				**Low-responder group**				
Variables	Median	Min	Max	N	Median		Min	Max	N

**At birth**									
Gestational age (weeks)	39	38	42	13	39		37	471	19
Height SDS	--1.49	--3.94	1.34	13	--1.93		--3.82	--0.18	19
Weight SDS	--1.90	--2.77	1.59	13	--1.20		--2.89	0.80	19
**Pretreatment**									
Delta height SDS during pretreatment yr	--2.84	--3.57	--2.24	11	--2.88		--4.13	--2.17	18
**At GH start**									
Gender (min = girls, max = boys)		2	11	13			4	15	19
AGA/SGA (min = AGA, max = SGA)		8	5	13			10	9	19
*Age (yrs)	5.84	3.90	10.53	13	9.31		3.32	11.96	19
Height SDS	--2.66	--3.84	--2.04	13	--2.84		--4.33	--1.59	19
Weight SDS	--2.51	--3.56	--1.72	13	--2.20		--4.11	--1.15	19
Father height SDS	--0.98	--1.59	2.12	13	--0.98		--2.95	0.85	19
Mother height SDS	--0.80	--2.04	1.15	13	--1.58		--2.93	0.56	19
MPHSDS	--0.54	--1.81	0.55	13	--1.21		--2.46	0.63	19
GH_max _AITT	21.86	20.20	48.07	13	28.75		18.20	63.86	17
*GH_max _24 h	23.81	23.40	39.68	6	53.71		27.12	87.78	12
Diff MPH SDS	--2.13	--3.73	--0.85	13	--1.67		--2.81	--0.34	19
IGF-I SDS	--1.02	--2.56	1.34	11	--0.98		--4.62	1.08	17
*Leptin (ng/mL)	3.96	2.14	5.93	12	2.40		1.21	4.30	17
**During treatment**									
*GH dose (U/kg/day)	0.11	0.09	0.12	13	0.10		0.09	0.11	19
*Change in height SDS 1st yr	0.96	0.89	1.26	13	0.42		0.25	0.51	19

AITT, arginine – insulin tolerance test; GH_max_, maximum peak of GH secretion; IGF-I, insulin-like growth factor I; AGA, appropriate for gestational age; SGA, small for gestational age; MPH midparental height; diffMPHSDS, difference in height SDS of the child versus its midparental height SDS

0.1 U GH/kg/day = 33 μg/kg/day

The references used for SDS calculations are [8] for height and weigh and [9] for IGF-I.

* indicates p < 0.05 between groups.

All patients were of Caucasian origin and children with dysmorphic syndromes or chronic diseases, were excluded from the study. Data analysis included the following variables; GH_max _after AITT, spontaneous GH secretion level over a 24 h period, IGF-I level, IGF-binding protein 3 (IGFBP-3) level and leptin level [6]. The references used for SDS calculations are [8] for height and weigh and [9] for IGF-I. The children received daily injections of 33 μg/kg recombinant human GH produced from the companies with GH products registered in Sweden.

### Study design

Blood samples were taken at two different time-points. The first sample was taken before the start of GH treatment and the second 1 year after the start of GH treatment. All samples were stored at -70°C for between 4 and 20 years and were not thawed until the time of analysis.

### Ethics

The study was performed in agreement with the Helsinki Declaration and approved by the Ethical Committee at the Sahlgrenska Academy, University of Gothenburg (no 270-88 and 71516-05). Informed consent was obtained from the parents of each child and from the child if old enough.

### SELDI-TOF serum protein profiling

Serum samples were thawed, denaturated and fractionated using serum anion exchange beads according to protocols provided by Bio-Rad Laboratories, Hercules, CA. Preliminary analyses were performed on all serum fractions from four low and four high responders, using weak anion-exchange (CM10), immobilized metal affinity capture (IMAC30) and reversed phase (H50) arrays (Bio-Rad Laboratories) in order to determine which conditions should be used for analysis of the whole study group. Serum fraction 6 run on CM10 arrays was identified as the most promising condition.

CM10 arrays were equilibrated twice with binding buffer (150 μL; 100 mM NaAcetate, pH 4.0). 10 μL sample and 90 μL binding buffer were applied on spots and mixed at room temperature using a DPC MicroMix 4 for 1 h for protein binding. Duplicate samples were analyzed for each patient at each time point. Arrays were washed three times in binding buffer, rinsed twice in 1 mM HEPES, and air-dried. 0.6 μL of a 50 % solution of sinapinic acid (Bio-Rad Laboratories) in 0.5 % trifluoroacetic acid (Merck, Darmstadt, Germany) and 50% acetonitril (Merck) was then applied twice to each spot as a matrix.

Time-of-flight spectra were generated using a PBS IIc ProteinChip reader (Bio-Rad Laboratories). Instrument settings for analysis were optimized in the mass range of 2.3–20.0 kDa and data were averaged from 240 transients for each protocol. Samples were randomized and analyzed concurrently within 1 week by the same operator in order to minimize experimental variation. One reference serum sample was randomly applied on each array. The mass accuracy was calibrated in the molecular range of 5–18 kDa using external calibrators from Bio-Rad Laboratories. The same calibration equation was used for all samples.

### Data preprocessing

Data handling was performed using ProteinChip Data Manager (Bio-Rad Laboratories). All spectra were baseline-subtracted and normalized according to total ion current, using an external normalization coefficient. Settings for peak identification and clustering of peaks across multiple spectra were first pass signal-to-noise ratio (S/N) > 5 in 15% of all spectra and second pass S/N > 3, with a cluster mass window 0.3% of the mass.

Spectra were visually inspected and data for patients were excluded from further analysis if duplicated profiles differed or if the overall quality was low in one or both of the spectra (i.e. high noise, overall low peak intensity or a normalization factor exceeding 2). This process resulted in 33 valid peaks with mass/charge (m/z) values between 2.5 and 20.0 kDa. The average coefficient of variation (CV) for the 17 peaks detected in all of the reference samples was 18% (11–31%).

### Statistics

To identify peaks of potential interest, serum proteomic profiles for the high- and low-responder groups were compared before and after 1 year of treatment. Changes in maximum protein peak intensities during 1 year of GH treatment were also assessed.

Initially, ProteinChip Data Manager was used for calculation of p-values for single peaks. For paired statistics, ProteinChip Data Manager compared the mean values of peak clusters, by sample group, using the Wilcoxon signed-rank test.

### Multivariate statistics

Subsequent multivariate data analysis was performed with Matlab software (version 7.3.0 R2006b, The Mathworks) on the mean intensity levels of the duplicate samples.

### Cross-validated stepwise regression

Using stepwise regression, subsets of peaks were selected and assessed to examine the difference between high and low responders (before and 1 year after GH treatment) or the differences between pre-treatment and 1 year of GH treatment. Varying the input parameters of the stepwise regression generated a set of potential regression models. Only models where the final number of peaks selected was not more than 10% of the total number of samples were studied further. The best regression model was selected by tenfold cross-validation, where the tenfold cross-validation was repeated ten times in order to obtain more reliable estimates of the cross-validated R^2^.

### Relationships with other variables

The procedures described above were also applied to explore the relationships between the peaks, instrumental and clinical variables, such as early growth data, parental height, IGF-I levels, leptin levels and the predicted response from published GH prediction models.

### Rotated two-component PLS regression/PLS discriminant analysis

Partial least squares (PLS) regression was used to explore the relationships between the most predictive protein peaks. We selected between 2 and 15 of the peaks that had the highest correlation with the response variable and used the binary high/low-responder classification or the observed delta height SD scores after 1 year of GH treatment as the response variable. A two-component PLS regression was computed with two latent variables and then rotated in such a way that the first rotated component gave the ordinary least-squares regression on the response variable and the second rotated component had maximum residual variance for the predictor variables. If the response variable is a binary variable, the PLS regression is comparable to a PLS-discriminant analysis (PLS-DA), and in that case the first rotated component will give the discrimination function. The R^2 ^and the tenfold cross-validated R^2 ^values of the PLS regression are presented. For the PLS-DA we also present the cross-validated correct rate of classification in percentage (CVcorrect).

### Analysis of systematic errors

Principal component analysis (PCA), PLS regression and stepwise regression were used to analyze the impact of systematic errors on the results. For a few peaks there was a weak linear relationship between peak intensity and storage time. The influence of storage time on peak intensity was corrected for using linear regression. Two peaks (m/z values of 4.149 and 4.182 kDa) had a non-linear relationship with storage time and were therefore excluded from further data analysis.

### Protein identification

#### Immunodepletion

Fractionated serum was immunodepleted using rabbit anti-transthyretin (DAKO, Glostrup, Denmark) or anti-apolipoprotein A-II (GenScript Corporation, Piscataway, NJ) antibodies and dynabeads (Invitrogen, Carlbad, CA) coated with anti-rabbit IgG. Dynabeads were washed twice in 1 mL PBS (PAA Laboratories GmbH, Pasching, Austria) and resolved in 0.5 mL PBS. 4 μg of antibody was added per 50 μL dynabeads and incubated over night at 4°C on a rotation mixer (REAX 2, Heidolph, Schwabach, Germany). Unbound antibodies were removed by washing twice in 1 mL PBS. The beads were resolved in 50 μL PBS, and 20 μL of fractionated serum was then added followed by incubation for 1 h at 4°C on a rotation mixer, followed by two washes in 1 mL PBS. The depleted serum was analyzed on CM10 arrays.

#### In-gel protein digestion

In-gel protein digestion with trypsin was, with some minor modifications, performed as described by Shevchenko et al. [10].

#### Nanoflow liquid chromatography/tandem MS (LC -MS/MS) Fourier transform ion cyclotron MS (FT/ICR MS)

Two-microliter sample injections were made with an HTC-PAL autosampler (CTC Analytics AG, Zwingen, Switzerland) connected to an Agilent 1100 binary pump (Agilent Technologies, Palo Alto, CA, USA). The peptides were trapped on a precolumn (45 × 0.075 mm i.d.) packed with 3 μm C_18_-bonded particles and separated on a reversed phase column, 200 × 0.050 mm. Both columns are packed in-house with 3 μm Reprosil-Pur C_18_-AQ particles. A 40 min gradient 10–50% CH3CN in 0.2% COOH was used for separation of the peptides.

Nanoflow LC-MS/MS was performed on a hybrid linear ion trap-FTICR mass spectrometer (LTQ-FT, Thermo Electron, Bremen, Germany). The spectrometer was operated in data-dependent mode, automatically switching to MS/MS mode. MS-spectra were acquired in the FTICR, while MS/MS-spectra were acquired in the LTQ-trap. All the tandem mass spectra were searched by MASCOT (Matrix Science, London, UK) against the SwissProt 5.16 database. The search parameters were set to: MS accuracy 5 ppm, MS/MS accuracy 0.5 Da, one missed cleavage by trypsin allowed, fixed propionamide modification of cysteine and variable modification of oxidized methionine.

## Results

### Changes in protein expression profile in response to GH during the first year of treatment

**Figure 2:**
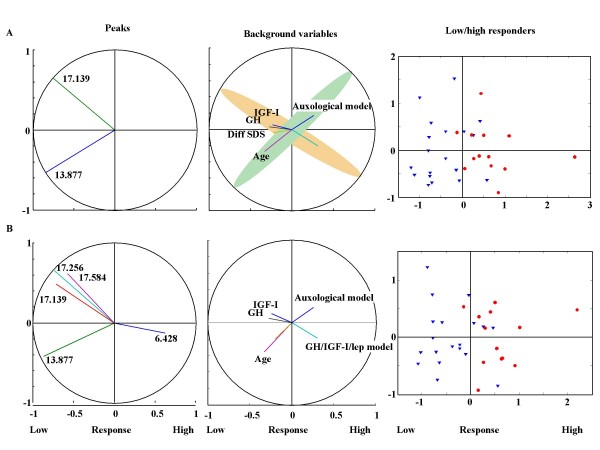
**Computerized selection of the most predictive peaks for discrimination between high and low responders to GH treatment**. The figure shows the rotated partial least squares regression using the 2 (A) and 5 (B) most predictive protein peaks. In all analyses, changes in the peak intensity during the first year of GH treatment are used. The left panels demonstrate which peaks are selected and the degree to which they correlate with the first dimension (low/high response on the x-axis). The first dimension (Predicted response) is a weighted sum of the selected peaks and gives the best prediction of the binary low/high-response variable. A positive correlation with the first dimension indicates that the maximum peak intensity has increased more for the high responders than low responders during 1 year of treatment, whereas a negative correlation indicates that the maximum peak intensity has increased more for the low responders. The second dimension is a residual component. Residual, in the sense that it has no correlation with the response variable and, component, in the sense that it of all the selected predictor variables it explains the maximum variance. The use of this dimension facilitates the discovery of clusters of related peaks, or peaks conveying comparable information. In the middle panels, correlations of dimensions 1 and 2 with phenotype are shown. The right panels illustrate the discrimination of the individual low and high responders using the selected peaks. Low responders are shown as triangles; high responders as circles.

In all data analyses, both the binary variable low versus high responder, and the continuous variable first year delta height SD, were used as outcome variables. This produced similar results. For a number of the peaks identified, we found that the change in peak intensity during the first year of treatment correlated with GH responsiveness. Thus, these peaks could be used as biomarkers for classifying patients as good or poor responders to GH treatment. Using information from between 2 and 15 peaks resulted in an R^2 ^in the range of 0.47–0.51 and a CVcorrect in the range of 82–64 % (Table 2, Figure 2). The 15 most predictive peaks identified by the rotated two-component PLS regression analysis are shown in Table 2. Using information on the intensity changes of the peaks with m/z values of 13.877 and 17.139 kDa, 82% of the study subjects could be correctly classified as low or high responders, respectively. Including more than two peaks in the analysis did not improve classification.

**Table 2:** The most predictive peaks identified by rotated two-component PLS regression analysis.

Number of peaks	R^2^	CVcorrect	First year growth hormone responsiveness Peak m/z value (kDa)
15	0.51	64	17.584, 17.383, 17.256, 17.139, 13.788, 9.127, 8.820, 8.689, 6.833, 6.626, 6.474, 6.428, 4.401, 3.212 and 3.311

5	0.47	76	13.877, 17.139, 17.256, 17.584 and 6.428

4	0.47	78	13.877, 17.139, 17.256 and 17.584

3	0.47	81	13.877, 17.139 and 17.256

2	0.47	82	13.877 and 17.139

			Before treatment

15	0.32	59	3.311, 4.401, 4.463, 7.010, 8.689, 8.820, 9.709, 13.877, 14.040, 14.142, 15.132, 17.139, 17.256, 17.584, 28.067

5	0.23	63	14.040, 14.142, 17.139, 17.256, 28.067

4	0.24	65	14.142, 17.139, 17.256, 28.067

3	0.32	74	9.361, 14.040, 17.256

2	0.17	59	17.139, 17.256

The most predictive peaks identified by rotated two-component PLS regression analysis when 2, 3, 4, 5 and 15 peaks were included in the analysis. For each model the R^2^-value and correct rate of classification (CVcorrect) are presented. It is the peak intensity changes during the first year of GH treatment that are used in the analyses.

**Figure 3:**
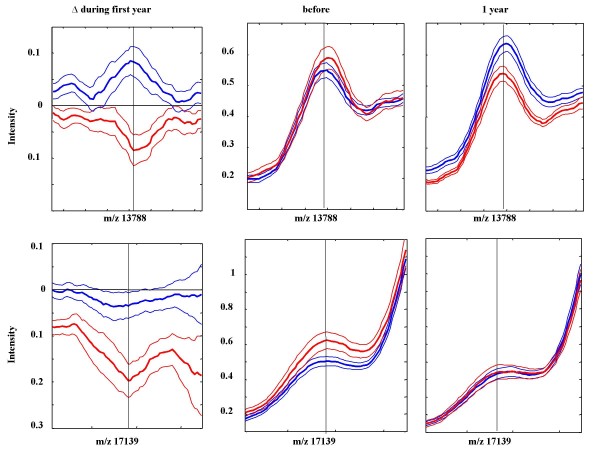
**Change in mean peak intensities**. Change in mean peak intensity (thick line) ± SEM during the first year of GH treatment (left panels), before the start (middle panels) after 1 year of treatment (right panels) for the 13.788 and the 17.139 kDa peaks that were most predictive for discrimination between children with a high (red) or low (blue) growth response to treatment.

The middle panel in Figure 2A shows that auxological data correlated with changes in the maximum intensity of the 13.877 kDa peak, and that variables associated with the GH/IGF-I axis, including IGF-I levels, leptin levels and GH_max_24 h, correlated with changes in the intensity of the 17.139 kDa peak. Mean spectra of the 13.877 and 17.139 kDa peaks for high and low responders were created (Figure 3). As shown in the figure, in the high-responder group the intensities of both peaks decreased during the first year of GH treatment. In the low-responder group, the intensity of the 13.877 kDa peak was increased during the first year of treatment, whereas the intensity of the 17.139 kDa peak was unchanged during treatment.

### Protein profiles before start of GH treatment

We were then interested to explore whether protein expression profiles could also be used to predict the growth response and level of GH responsiveness before start of treatment. Using the same statistical methods as described above, we found that information from between 2 and 15 peaks resulted in an R^2 ^in the range of 0.17–0.32 and a CVcorrect in the range of 74–59 % (Table 2). The best model for prediction of GH response before start of treatment was obtained when the 3 peaks with m/z values of 9.361, 14.040 and 17.256 kDa were included in the model (R^2 ^= 0.32, CVcorrect = 0.74).

### Identification of selected peaks

After identifying specific peaks that could be used to discriminate between high and low responders to GH treatment, we wanted to identify the proteins representing the most predictive peaks. From the consistency of the peak pattern in the spectra, the peaks with m/z values around 14 kDa were recognized as different posttranslational modified forms of transthyretin; the 13.877 kDa peak was recognized as the unmodified form and the 14.040 kDa peak was recognized as the cysteinylated form. The 17.139 and 17.256 kDa peaks were recognized as dimers of apolipoprotein A-II (Apo A-II), the 8.609 and 4.401 kDa peaks were recognized as truncated forms of Apo A-II, and the 6.428 and 3.212 kDa peaks were recognized as truncated forms of Apo C-I. To verify the identities of the two most discriminating proteins we performed serum depletion experiments using specific antibodies and MS protein identification.

**Figure 4:**
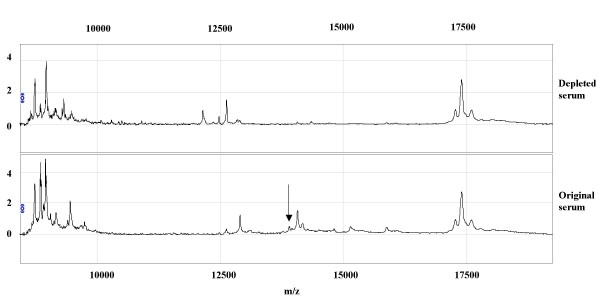
**Depletion of transthyretin from serum using specific anti-transthyretin antibodies**. The upper panel represents the depleted serum and the lower panel represents the original sera before depletion. The peak with the molecular mass of 13.877 kDa is indicated by an arrow.

An anti-human transthyretin antibody depleted both a 12.865 kDa peak, formerly identified as a fragment of transthyretin [11], and a group of peaks around 14 kDa, including the 13.877 and 14.040 serum peaks (Figure 4). Using the anti-Apo A-II antibody, we were not able to deplete any peaks. However, when the quality of the antibody was checked by SELDI-TOF we found very low amounts of full-length antibodies and high levels of light and heavy chains. Therefore, serum fraction 6 was separated on a 1D SDS PAGE and bands in the area around 17 kDa where cut out, trypsin digested and the resultant samples underwent MS identification. The result indicated that the samples were not pure. After common impurities like keratin had been excluded, there were eight potential proteins left for evaluation. The proteins were in the following order based on the number of assigned peptides: Apo A-II, serum amyloid A 4, hemoglobin subunit alpha, Apo A-I, hemoglobin subunit beta, transthyretin, histone H4 and albumin. Apo A-II had the highest number of assigned peptides. The MS identification results, together with the characteristic serum profile pattern, strongly suggest that the 17.139 and 17.256 kDa peaks represent Apo A-II.

## Discussion

The present study utilized a pharmaco-proteomic approach in order to identify novel biomarkers that can be used to predict growth in response to GH treatment in short prepubertal children with idiopathic short stature. Using this approach we identified two peaks that together enabled the correct classification of 82% of the study subjects as either good or poor responders to GH treatment. Through further analysis, using a combination of the specific peak patterns within the spectra, results from serum-depletion experiments and MS identification, we identified these peaks to represent transthyretin and Apo A-II.

One of the two most discriminatory peaks in the present study was the unmodified form of transthyretin, also known as prealbumin. In addition to its functions as a carrier of serum thyroxine and triiodothyronine and a transporter of retinol [12], transthyretin is known as a marker of nutrition. Studies have shown that in cases of malnutrition there is a poor growth response to GH [13]. Clinical studies also show that a high leptin level, a marker of adipocyte tissue mass, at start of GH treatment correlates with a better growth response [14]. The intensity of the transthyretin peak increased in the low-responder group and decreased in the high-responder group during GH treatment. Interestingly, GH administration has also been shown to increase serum levels of transthyretin in severely burned children and in adults after hepatectomy [15,16], suggesting a common mechanism between serious illness and low GH responsiveness. Our results are in line with a report showing that serum levels of transthyretin in rats were markedly decreased by hypophysectomy and further decreased after GH replacement [17]. Furthermore, transthyretin has been shown to play a role in type 1 diabetes. Patients with type 1 diabetes had lower serum levels of transthyretin than healthy controls. In addition, the transthyretin tetramer, in contrast to the transthyretin monomer, promoted insulin release [18].

The second most predictive peak was Apo A-II. This peak remained unchanged in the low responders, but decreased in the high responders after GH treatment. Apo A-II is the second most abundant protein component of high-density lipoprotein (HDL)-cholesterol but little is known about its role in HDL metabolism. GH plays a role in lipid and lipoprotein metabolism in man, increasing both the uptake and degradation of low-density lipoproteins [19]. A study of GH-deficient children suggested that GH had no effect on serum Apo A-II levels [20]. Experiments using mouse models have shown a role for Apo A-II in glucose homeostasis: Apo A-II-deficient mice showed improved insulin sensitivity, whereas transgenic mice over-expressing murine Apo A-II showed insulin resistance and obesity [21]. Apo A-II has also been suggested as a candidate gene in type 2 diabetes [22-24].

Using rotated two-component PLS regression and PLS discriminant analysis, we have shown that the protein peak corresponding to transthyretin correlates with auxological data as shown in Figure 2. Moreover, apolipoprotein levels appear to correlate with changes in the GH/IGF/leptin axis. This could partly explain the influence of nutrition on GH responsiveness. However, our results must be validated on an independent patient sample.

In this study we analyzed serum protein profiles from before the start of GH treatment and after 1 year of treatment. This time period was selected as growth response during 1 year has been shown to give an estimate of GH responsiveness, known as the response score. We were interested to see whether changes during this time period in the expression of the novel biomarkers identified could be of additional value in predicting long term prepubertal growth [25,26].

In order to predict the growth response to GH treatment, it is preferable to be able to use pre-treatment data. From the protein profiles before the start of treatment we were able to create a model based on the intensity levels of three peaks. However, this model was not as robust as the model for changes in response to GH treatment. The optimal time period needed to ascertain changes that may be of utility in predicting final height is certain different for different variables. For example, we know that short-term changes in serum IGF-I levels are a better predictor of growth response than serum levels of IGF-I before the start of treatment [27]. In this context, it would also be of interest to analyze short-term changes in protein profiles after the start of treatment, for example after 1–2 weeks.

SELDI-TOF is a complementary technology to other proteomic techniques like 2D gel electrophoresis; for our purposes, a major advantage is that SELDI-TOF is a high throughput technique and is therefore more suitable for analyzing a large quantity of samples than 2D gel electrophoresis. Furthermore, SELDI-TOF technology has an improved sensitivity in the mass range < 25 kDa, whereas 2D electrophoresis has a high resolution and sensitivity between 20 and 150 kDa. The reproducibility and reliability of the SELDI-TOF-MS proteomic system have been discussed [28-30]. Transit time, storage conditions, clotting time and tube type used can affect protein profiles [31,32], which has raised concerns about using samples from retrospective studies. However, proper handling of samples can minimize these shortcomings [33], and in the present study we took special precautions to ascertain standardization. Samples were stored at -70°C and were not thawed until used for analysis. They were randomized to each bioprocessor and the analyses were run within a week by the same operator. A reference sample was applied on each array. Using PCA, PLS regression and stepwise regression, we did not detect systematic errors correlated with non-GH-dependent factors or instrumental biases such as array or spot number, but for some peaks we found a weak linear relationship between peak intensity and storage time, which was adjusted for in all data analyses. Two peaks with a non-linear relationship to storage time were excluded from further analysis. The average CV of 18% for 17 peaks in the reference samples was in the expected range for this method [34].

In order to find peaks related to GH responsiveness we used an integrated strategy, combining the results from cross-validated stepwise regressions and PLS regressions. The best distinction between good and poor responders over the first year of GH treatment was obtained using two peaks; adding more peaks into the model increased background noise.

Reciprocal relationships between the most predictive peaks were visualized with rotated PLS regression to explore the formation of clusters of peaks containing comparable information. As the target response variable, we explored the prediction of the binary low/high response and the first year growth response in height SD score; results were similar.

Only one previous study has utilized SELDI-TOF proteomics to search for GH-dependent biomarkers [35]. In this study the aim was to identify novel biomarkers of GH administration in adult athletes. A peak of 15.1 kDa was identified as the most effective classifier of subjects receiving GH. This peak was identified as hemoglobin α-chain (HbA1). We did not identify this peak to be influenced by GH treatment in short children, possibly due to the different study groups and different ProteinChip surfaces that were utilized in the two studies.

From the information of isoelectric points, the known GH-dependent proteins IGF-I, IGFBP-3, leptin and insulin are not expected to be identified in protein profiles from serum fraction 6 on CM10 arrays. IGF-I is expected to be detected in fraction 1, and IGFBP-3, leptin and insulin are expected to be detected in fraction 3. In the pilot study of four good and four poor responders, performed with all fractions on three array surfaces, a 7.643 kDa peak corresponding to the mass of IGF-I, was detected on CM10 arrays in fraction 1. This peak was up-regulated by 70% during the first year of GH treatment in the high-responder group, whereas there was no significant change in peak intensity in the low-responder group.

In summary, we have shown that analysis of serum protein expression patterns can be a useful method for identifying novel markers of GH responsiveness. In future, knowledge obtained using broad exploratory techniques, could possibly be used in the development of new clinical tools for diagnosis and treatment.

## Conclusion

In this study we show that a pharmaco-proteomic approach can be used in order to identify novel biomarkers that can be used to discriminate between good and poor responders to GH treatment among short prepubertal children with ISS. Optimal results were obtained when changes in peak intensity during first year of treatment were analyzed. Using this approach we identified two peaks that together enabled the correct classification of 82% of the study subjects as either good or poor responders to GH treatment. Through further analysis, using a combination of the specific peak patterns within the spectra, results from serum-depletion experiments and MS identification, these peaks were identified to represent transthyretin and Apo A-II. These results suggest that these proteins may have a role in determining GH sensitivity.

## Abbreviations

GH: growth hormone; GHD: growth hormone-deficient; ISS: idiopathic short stature; SDS: standard deviation score; SELDI-TOF-MS: surface-enhanced desorption/ionization time-of-flight mass spectrometry; Apo A-II: apolipoprotein A-II; IGF-I: insulin-like growth factor I; GH_max_: maximum peak of GH secretion; AITT: arginine-insulin tolerance test; IGFBP-3: IGF-binding protein 3; S/N: signal-to-noise ratio; m/z: mass/charge; CV: coefficient of variation; PCA: principal component analysis; PLS: partial least squares; PLS-DA: PLS-discriminant analysis; CVcorrect: correct rate of classification in percentage; MS LC -MS/MS: liquid chromatography/tandem MS; FT/ICR MS: Fourier transform ion cyclotron MS; HDL: high-density lipoprotein; HbA1: hemoglobin α-chain.

## Competing interests

KAW declares that she has an unrestricted research grant from Pharmacia/Pfizer. AFMN works for Muvara, Multivariate Analysis of Industrial and Research Data Statistical Consultation, The Netherlands. GH, BA, JD, and ZH declare that they have no competing interests.

## Authors' contributions

GH, BA, AFMN, JD, ZH and KAW have all given substantial contribution to conception and design, analysis and interpretation of the data. GH and BA have designed and carried out all the SELDI-TOF and additional experiments. AFMN performed all of the statistical analyses. GH, BA, AFMN, JD, ZH and KAW have all been involved in drafting the manuscript and have revised it critically for important intellectual content.
